# Synthesis and Evaluation of Ubiquitin–Dioxetane Conjugate as a Chemiluminescent Probe for Monitoring Deubiquitinase Activity

**DOI:** 10.1021/acs.bioconjchem.1c00413

**Published:** 2021-09-22

**Authors:** Sara Gutkin, Satish Gandhesiri, Ashraf Brik, Doron Shabat

**Affiliations:** † School of Chemistry, Raymond and Beverly Sackler Faculty of Exact Sciences, Tel Aviv University, Tel Aviv 69978, Israel; ‡ Schulich Faculty of Chemistry, Technion-Israel Institute of Technology, Haifa 3200008, Israel

## Abstract

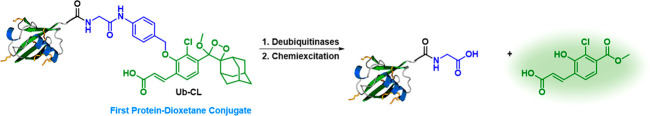

The removal of ubiquitin (Ub) from a modified protein or Ub chain is a process that occurs regularly by the ubiquitin–proteasome system. This process is known to be mediated by various deubiquitinating enzymes (DUBs) in order to control the protein’s half-life and its expression levels among many other signaling processes. Since the function of DUBs is also involved in numerous human diseases, such as cancer, there is an obvious need for an effective diagnostic probe that can monitor the activity of these enzymes. We have developed the first chemiluminescence probe for detection of DUBs activity. The probe was prepared by conjugation of the chemically synthesized C-terminally activated Ub(1-75) with a Gly-enolether precursor. Subsequent oxidation, under aqueous conditions, of the enolether conjuagate with singlet-oxygen furnished the dioxetane probe **Ub-CL**. This synthesis provides the first example of a dioxetane–luminophore protein conjugate. The probe’s ability to detect deubiquitinating activity was successfully validated with three different DUBs. In order to demonstrate the advantage of our new probe, comparison measurements for detection of DUB UCH-L3 activity were performed between the chemiluminescent probe **Ub-CL** and the well-known **Ub-AMC** probe. The obtained data showed significantly higher S/N, for probe **Ub-CL** (>93-fold) in comparison to that observed for **Ub-AMC** (1.5-fold). We anticipate that the successful design and synthesis of the turn-ON protein–dioxetane conjugate probe, demonstrated in this work, will provide the insight and motivation for preparation of other relevant protein–dioxetane conjugates.

Ubiquitination is a reversible post-translational modification that involves the covalent attachment of ubiquitin (Ub) monomer or Ub chains to a target protein.^1^ This process regulates the degradation of cellular proteins by the Ub-proteasome system (UPS) and controls a protein’s half-life, therefore affecting numerous signaling pathways.^2,3^ While the conjugation of Ub to a target protein is performed by the ubiquitination enzymes (E1, E2, and E3), the removal of Ub from a ubiquitinated protein is mediated by a family of deubiquitinating enzymes (DUBs).^4−6^ Since the function of DUBs is also involved in many human diseases, there is an obvious need for an effective diagnostic probe that can monitor the activity of these enzymes.^7,8^ Such a probe is ordinarily used to screen small molecule inhibitors against DUBs for studying DUB activities and drug development.^9^ Indeed, optical molecular probes for detection of DUB activity were developed years ago.^10−13^ The detection mode of these probes is usually based on fluorescence, where 7-amino-4-methylcoumarin (AMC) is used as a fluorescent dye.^14−16^ Such a ubiquitin–AMC conjugate exhibits a typical turn-ON fluorescence response, following the DUB-mediated hydrolysis.

Chemiluminescence modality has an inherent advantage over fluorescence, since irradiation by an external light source is not required.^17,18^ As a result, there is no interference by autofluorescence, and the obtained background signal is extremely low.^19^ Among the known chemiluminescent luminophores, the triggerable phenoxy-dioxetanes, discovered by Paul Schaap in 1987, are commonly used for chemiluminescent probe design.^20,21^ Four years ago, a major breakthrough was achieved by our group, with the development of new-generation phenoxy-dioxetane luminophores.^22,23^ These new dioxetane chemiluminescent luminophores exhibit up to 3000-fold enhancement in light-emission quantum yield (ΦCL) under physiological conditions.^24,25^ Our group and others have utilized these dioxetane luminophores to prepare chemiluminescent probes for the detection and imaging of various enzymes and chemical analytes.^26−28^

The most effective chemiluminescent probes, in terms of sensitivity and signal-to-noise ratio, were obtained by masking the phenolic group of the luminophore with peptide substrates through a 4-aminobenzyl alcohol self-immolative linker.^29,30^ Such probes produce extremely low background signal, due to their high stability toward spontaneous hydrolysis.^31^ The enzymatic responsive group used to mask the phenoxy-dioxetane luminophores were all composed of small molecules or short peptides.^32−34^ A responsive group based on a full protein substrate, attached to the dioxetane luminophore, has never been demonstrated. Here, we report the design, synthesis, and evaluation of a new chemiluminescent probe, based on a ubiquitin–dioxetane conjugate, for efficient detection of DUB activity.

The general molecular structure and chemiexcitation disassembly pathway of the DUB chemiluminescent probe **Ub-CL** is presented in Figure 1. Probe **Ub-CL** is composed of the protein sequence Ub(1-76)-X, where X is the NH_2_ group of the self-immolative linker, *p*-amino-benzyl-alcohol (PABA). Proteolytic cleavage of the specific peptide bond between Gly^76^ and the PABA linker, followed by 1,6-elimination, releases the phenoxy-dioxetane luminophore **I**. This phenoxy-dioxetane then undergoes rapid chemiexcitation disassembly to produce benzoate **II** and a green photon.

**Figure 1:**
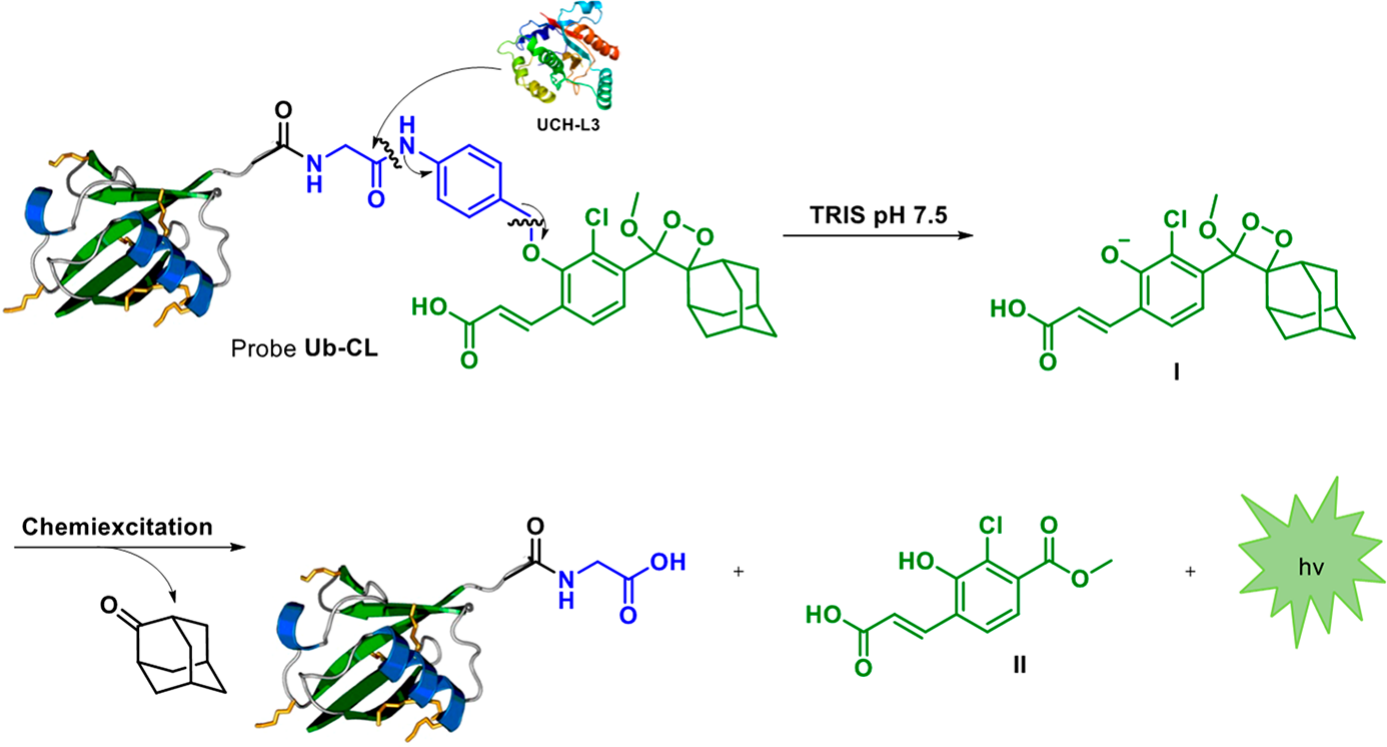
Molecular structure and chemiexcitation disassembly pathway of DUB chemiluminescent probe **Ub-CL**.

Phenoxy-dioxetane probes are regularly synthesized through oxidation of the corresponded enolether precursor by singlet oxygen. Therefore, we initially synthesized an enolether derivative, conjugated with the ubiquitin segment Ub(1-75). The chemical synthesis of conjugate Ub-Enolether **3** is presented in Figure 2. Fmoc-Gly was coupled with 4-aminobenzyl-alcohol to generate amide **1a**. Iodination of the benzylic position of **1a** with sodium iodide and trimethylsilyl chloride yielded benzyl-iodide **1b**. The latter was reacted with previously synthesized phenol **1c** under mild basic conditions to afford ether **1d**. The allyl and Fmoc protecting groups in compound **1d** were removed by Pd(PPh_3_)_4_. Subsequent addition of piperidine resulted with removal of the Fmoc protecting group to generate Enolether **2**. Next, the amine functional group of Enolether **2** was coupled with the chemically synthesized Ub(1-75)-MeNbz^35^ (compound **1**, see Supporting Information for synthesis) to yield the desired ubiquitin–enolether conjugate Ub-Enolether **3**.

**Figure 2:**
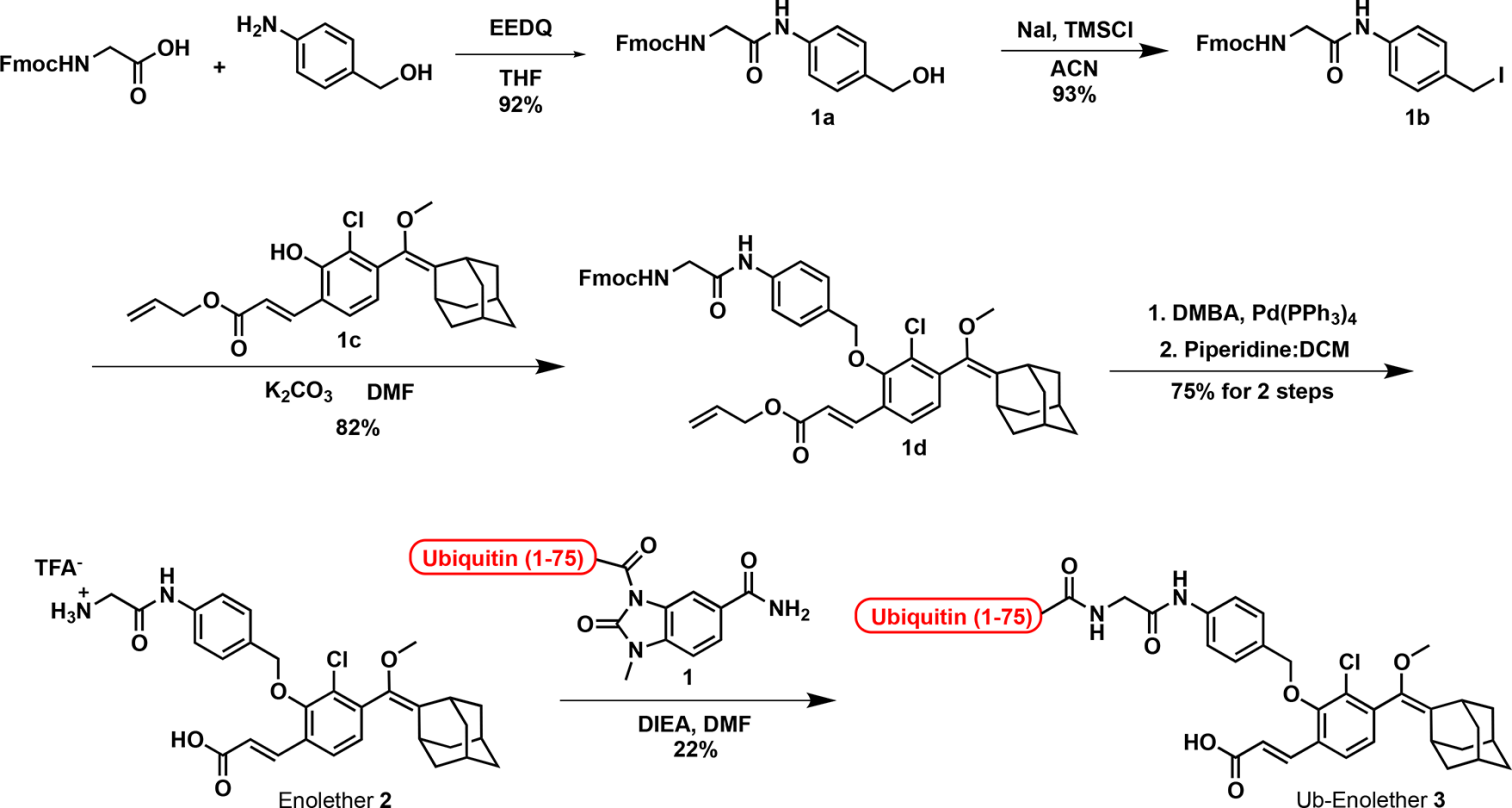
Synthetic route for the preparation of conjugate Ub-Enolether **3**.

With Ub-Enolether **3** in hand, we sought oxidation conditions, appropriate for enolether–protein conjugates, which can be performed by singlet oxygen.^36,37^ Since singlet oxygen is a highly reactive reagent, that undergoes quenching to some extent, by polar solvents like water,^38^ the oxidation procedure of the enolether precursor is usually performed in a nonpolar organic solvent like methylene chloride. The enolether precursor of probe **Ub-CL** is composed of the Ub protein, conjugated with the enolether small molecule. Such a protein conjugate can be mainly solubilized in aqueous solvents. Therefore, we initially sought to develop a procedure aimed to establish oxidation conditions for general enolethers, by singlet oxygen, in aqueous solvent, which can also be applied to other similar systems.

Enolether **2** was selected as a model compound for developing oxidation conditions in the aqueous solvent, PBS 7.4. Thus, the oxidation of Enolether **2** was evaluated in PBS 7.4, using polystyrene-bound Rose Bengal as a photosensitizer, and irradiation with white light, while oxygen is bubbled through the reaction solvent. The reaction progress was monitored by RP-HPLC (Figure 3A). Within 45 min under such conditions, more than 50% of Enolether **2** was oxidized to form the desired Dioxetane **2a**. In addition, gradual decomposition of the dioxetane to its corresponded Benzoate **2b** is also observed. After keeping the reaction time of 60 min, Dioxetane **2a** appeared as the major product of the reaction. Further extending the reaction time resulted in substantial formation of Benzoate **2b**. Therefore, oxidation under these conditions for 60 min yielded the optimal results in our hands.

**Figure 3:**
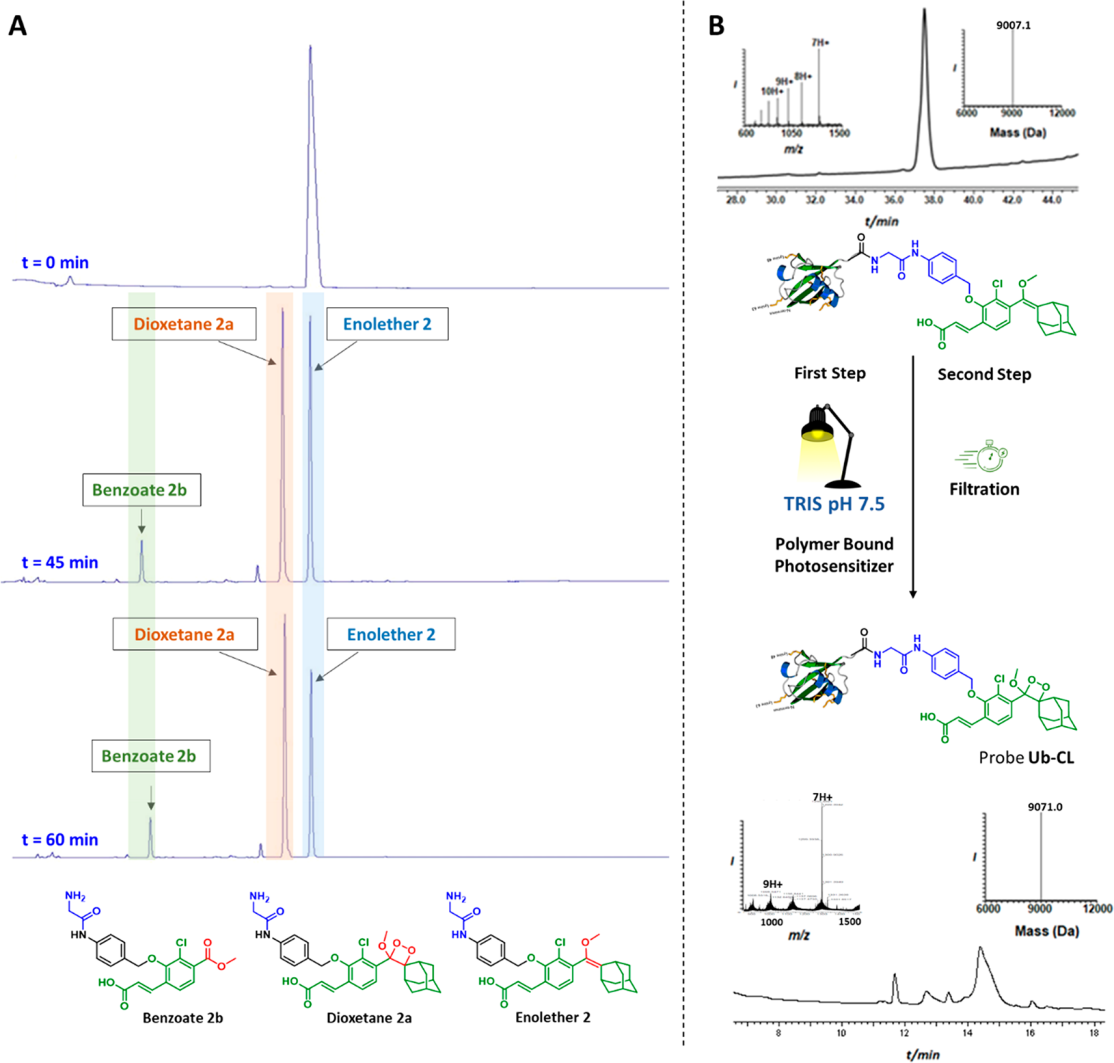
(A) RP-HPLC chromatograms showing the reaction progress, over 60 min, for oxidation of Enolether **2** to Dioxetane **2a** in PBS 7.4 as a solvent, polystyrene-bound Rose Bengal as a photosynthesizer, oxygen bubbling and irradiation with white light. The reaction progress was monitored at wavelength of 285 nm. (B) Oxidation of Ub-Enolether **3** by singlet oxygen to its corresponded dioxetane, probe **Ub-CL** in TRIS buffer, pH 7.5. The Mass obtained for dioxetane probe **Ub-CL** (9071) is suitable to oxidation of the S methionine group to SO_2_.

The establishment of conditions for the oxidation of enolethers to dioxetanes with singlet oxygen, under aqueous conditions, is of high importance, since protein–enolether conjugates are likely to be not soluble/compatible in/with organic solvents. Applying the established oxidation conditions on Ub-Enolether **3**, and subsequent filtration of the polystyrene-bound Rose Bengal photosensitizer, afforded the desired probe **Ub-CL** (Figure 3B). The obtained probe was used for measurements, in its current status, with no further purification steps.

With probe **Ub-CL** in hand, we sought to evaluate its ability to detect the catalytic activity of various DUBs. The probe was incubated in a suitable buffer and its chemiluminescence light emission profile was measured with three different DUBs: UCH-L3, UCH-L1, and USP-2 (Figure 4). Probe **Ub-CL** in the presence of USP-2 showed only slight light emission enhancement (2.5-fold), but still significantly higher than the background signal (Figure 4C). Remarkably, the total light emission signal produced by **Ub-CL** upon incubation with UCH-L1 (Figure 4B) and UCH-L3 (Figure 4A) was significantly higher than the background signal (in the absence of the DUB), with S/N values of 45 and 300, respectively. The substantially higher catalytic activity observed for UCH-L3 DUB, toward activation of probe **Ub-CL**, was observed before with other assays.^10,39,40^ Nevertheless, probe **Ub-CL** was able to clearly detect the activity of all three DUBs evaluated in this assay. It appears that probe **Ub-CL** is poorly turned over by USP-2; thus, this probe may be best suited for the UCH family DUBs.

**Figure 4:**
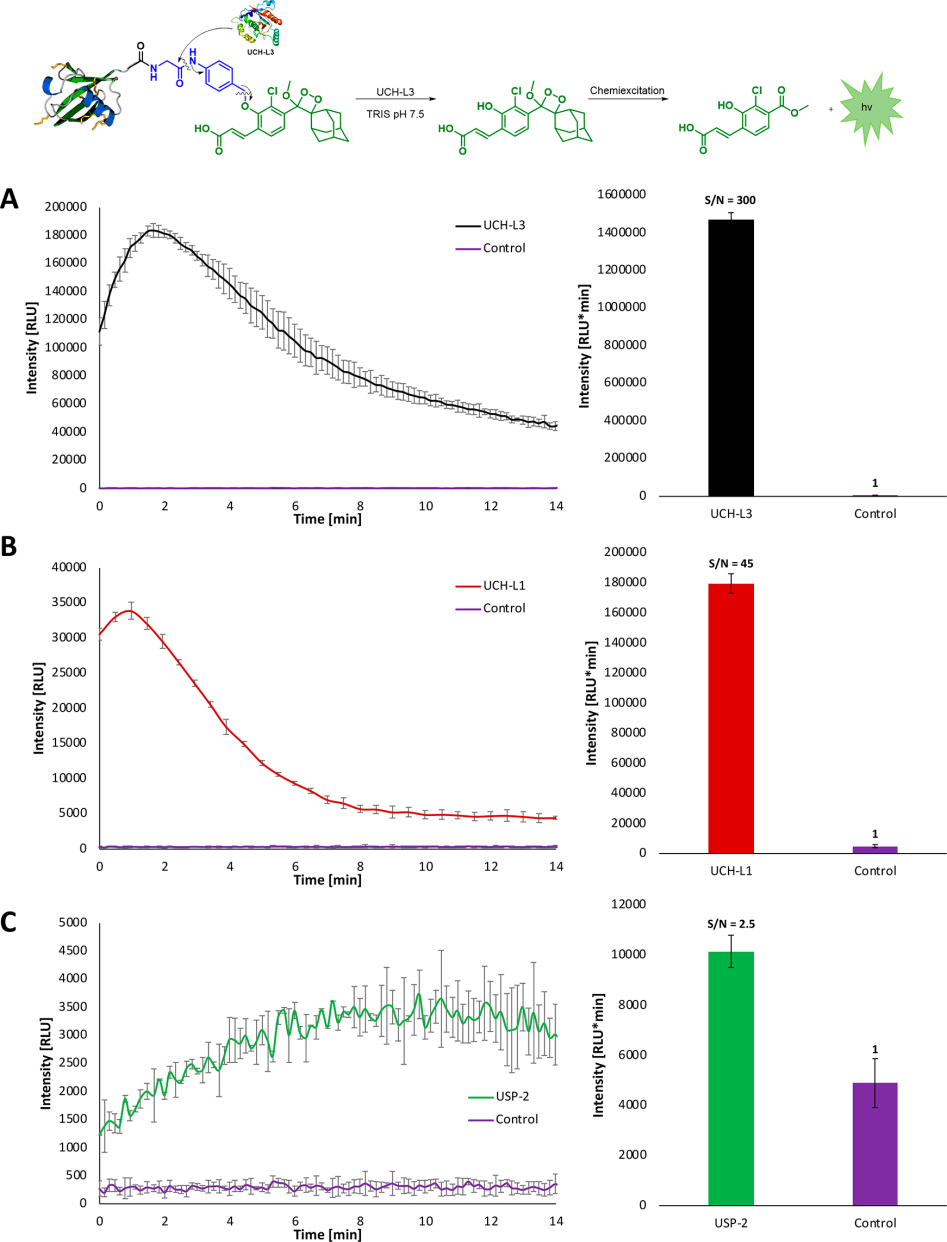
Chemiluminescence kinetic profile (left) and total light emission (right) of probe **Ub-CL** [10 μM] in TRIS PH 7.5, DTT [0.2 mM] with and without (A) UCH-L3 [0.8 nM], (B) UCH-L1 [50 nM], and (C) USP-2 [12 nM]. The total light emission was measured over 15 min at 37 °C. Error bars represent the mean of three different replicate measurements.

As mentioned above, turn-ON chemiluminescent probes have an inherent advantage over fluorescent probes. The request for an external light excitation source in fluorescence generates a substantial noise signal. In chemiluminescence, the excited state of the emitter is formed through breakage of energetic chemical bonds. When the molecule has high chemical stability, this mode of excitation practically produced zero noise signal. In order to demonstrate the advantage of our DUB chemiluminescent probe, we performed comparison measurements of the S/N ratio produced by probe **Ub-CL**, and the commercially available fluorescent probe **Ub-AMC**. The probes were incubated with and without DUB UCH-L3 in TRIS pH 7.5, under similar conditions, and the produced optical signal was measured over 25 min (Figure 5). Expectedly, both probes produced a typical turn-ON response, upon reaction with UCH-L3, for either the chemiluminescence or the fluorescence mode of action. However, the background signal produced by fluorescent probe **Ub-AMC** is considerably higher than the background signal observed for our chemiluminescent probe **Ub-CL**. In addition, probe **Ub-CL** exhibited a faster response to UCH-L3, with a signal intensity of up to 93-fold higher than the signal intensity without the DUB. In contrast, probe **Ub-AMC** produced only a 1.5-fold increase over the background signal without the DUB at a similar time slot. The significantly higher signal-to-noise ratio, obtained for probe **Ub-CL**, clearly demonstrates the superior detection capability of the chemiluminescence modality over a fluorescent one.

**Figure 5:**
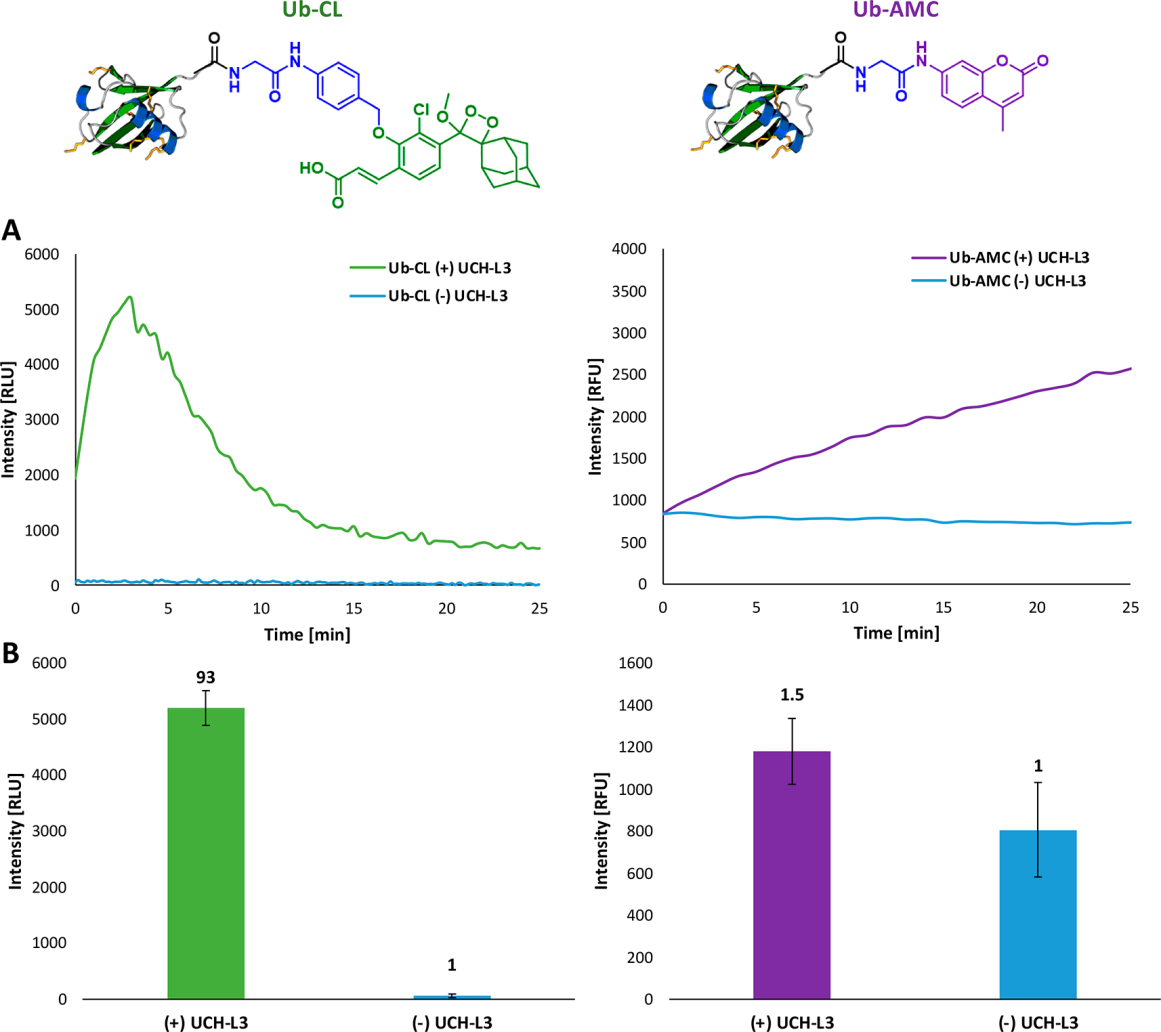
(A) Chemiluminescence (left) and fluorescence (right) kinetic profiles of **Ub-CL** [50 nM] and **Ub-AMC** [1 μM] in TRIS pH 7.5, DTT [0.2 mM] at 37 °C with and without UCH-L3 [0.8 nM]. (B) Signal to noise ratios obtained for probes **Ub-CL** (left) and **Ub-AMC** (right) with and without UCH-L3. The values were calculated at peak-max for chemiluminescence, after 3 min, since the starting measurement. Error bars represent the mean of three different replicate measurements.

The dioxetane–Ub conjugate, described herein, acts as a turn-ON chemiluminescent probe for detection of DUBs activity. The activation mechanism is based on the catalytic cleavage of Ub at a specific site, and results in the release of a phenoxy-dioxetane luminophore that undergoes rapid chemiexcitation to emit light. As shown in Figure 3, the oxidation of the Ub-enolether precursor with singlet oxygen, under aqueous conditions, was incomplete and also generates some benzoate decomposition product. However, unlike the fluorescence assay, in the chemiluminescence modality, such side products do not produce any noise signal. Thus, the obtained dioxetane–Ub conjugate can directly be used, without further purification, after removal of the polymer-immobilized photosensitizer by filtration. The exact concertation of the active dioxetane species of the **Ub-Cl** probe can be extrapolated by a light-emission calibration curve, obtained for an analogous known chemiluminescent probe. Importantly, the dioxetane functional group of the Ub conjugate was found to be highly stable over several days of storage (see Figure S7 in the Supporting Information), and the light-emission signal, produced by the dioxetane–ubiquitin probe, has remained quantitively similar over the evaluated time period.

In summary, we have developed the first chemiluminescence probe for detection of DUBs activity. The probe was prepared by conjugation of the chemically synthesized C-terminal of Ub^1–75^ protein with the NH_2_-Gly-enolether precursor. Subsequent oxidation, under aqueous conditions, of the enolether precursor with singlet oxygen furnished the synthesis dioxetane probe **Ub-CL**. This synthesis provides the first example of a dioxetane–luminophore conjugate with a protein. The probe ability to detect DUB’s activity was successfully validated with three different DUBs. In order to demonstrate the advantage of our new probe, comparison measurements for detection DUB UCH-L3 activity were performed between the chemiluminescent probe **Ub-CL** and a commercially available fluorescent probe **Ub-AMC**. The obtained data showed significantly higher S/N, observed for probe **Ub-CL** (93-fold) in comparison to that observed for probe **Ub-AMC** (1.5-fold). The successful synthesis and demonstration of this protein–dioxetane conjugate example, as a turn-ON probe, open a door for preparation of other relevant protein–dioxetane conjugates, such as those for Ub-like modifiers and for other biomacromolecules, e.g., nucleic acids.

## Supporting Information Available

Synthetic procedures, characterization data (NMR and MS) for all new compounds and chemiluminescence control experiments (PDF)
