# Supplementary material for: Synthesis and Evaluation of Ubiquitin–Dioxetane Conjugate as a Chemiluminescent Probe for Monitoring Deubiquitinase Activity
Source: Bioconjug Chem. 2021 Sep 22;32(10):2141–7. doi: 10.1021/acs.bioconjchem.1c00413 (PMC8589252; doi:10.1021/acs.bioconjchem.1c00413)
Supplement: Supplementary file 1 — bc1c00413_si_001.pdf [file bc1c00413_si_001.pdf]

Synthesis and Evaluation of Ubiquitin-Dioxetane Conjugate as a Chemiluminescent Probe for Monitoring Deubiquitinase Activity

Sara Gutkin[a,#], Satish Gandhesiri[b, #], Ashraf Brik[b]* and Doron Shabat[a]*

[a]School of Chemistry, Raymond and Beverly Sackler Faculty of Exact Sciences, Tel Aviv University, Tel Aviv 69978 Israel. [b]Schulich Faculty of Chemistry, Technion-Israel Institute of Technology, Haifa, Israel.

[#] These authors contributed equally

Supporting Information

Table of Contents

Synthetic Schemes and Experimental Procedures ……………………………………………………………2-8

Supplementary Figures ………………………………………………………………………………………………9-13

NMR spectra ……14-17

References………18

Synthetic Schemes and Experimental Procedures

General methods.

All reactions requiring anhydrous conditions were performed under an Argon atmosphere. All reactions were carried out at room temperature unless stated otherwise. Chemicals and solvents were either A.R. grade or purified by standard techniques. Thin layer chromatography (TLC): silica gel plates Merck 60 F254: compounds were visualized by irradiation with UV light. Column chromatography (FC): silica gel Merck 60 (particle size 0.040-0.063 mm), eluent given in parentheses. Reverse-phase high pressure liquid chromatography (RP-HPLC): C18 5u, 250x4.6mm and Waters, CSH C18, 3.5u, 4.6 × 150 mm , eluent given in parentheses. Preparative RP-HPLC: C18 5u, 250x21mm and Waters, C18, 10u, 250 × 19 mm, eluent given in parentheses. Semi-preparative HPLC was performed on a Thermo instrument (Dionex Ultimate 3000) using Phenomenex Jupiter C18 10u, 300 Å, 250 × 10 mm column. ^{1}H-NMR spectra were measured using Bruker Avance operated at 400MHz. ^{13}C-NMR spectra were measured using Bruker Avance operated at 100 MHz. Chemical shifts were reported in ppm on the δ scale relative to a residual solvent ($CDCl_3$: δ = 7.26 for ^{1}H-NMR and 77.16 for ^{13}C-NMR, DMSO-d_6: δ = 2.50 for ^{1}H-NMR and 39.52 for ^{13}C-NMR). Mass spectra were measured on Waters Xevo TQD, LCQ Fleet Ion Trap (Thermo Scientific) and Xevo G2-XS QTof (Waters). All calculated masses have been reported as an average isotope composition. Buffer A: 0.1% TFA in water; buffer B: 0.1% TFA in acetonitrile. Chemiluminescence was recorded on Molecular Devices Spectramax i3x. Fluorescence was recorded on Tecan infinite 200 Pro. All general reagents, including salts and solvents, were purchased from Sigma-Aldrich. Light irradiation for photochemical reactions: LED PAR38 lamp (19W, 3000K). Peptides were prepared by SPPS either manually in Teflon filter fitted syringes (purchased from Torviq) or by using an automated peptide synthesizer (CS336X, CSBIO). Analytical grade N, N-dimethylformamide was purchased from Biotech. Resins were purchased from Creosalus, protected amino acids were purchased from GL Biochem and activating reagents were purchased from Luxembourg Bio Technologies.

Abbreviations.

ACN- Acetonitrile, **DCM**- dichlorometane, **DIEA**- N,N-Diisopropylethylamine, **DMF**- N,N' -Dimethylformamide, **DMBA**- Dimethylbarbituric acid, **EEDQ**- N-Ethoxycarbonyl-2-ethoxy-1,2-dihydroquinoline, **EtOAc**- Ethylacetate, **HATU-** 1-[Bis(dimethylamino)methylene]-1H-1,2,3-triazolo[4,5-b]pyridinium3-oxid hexafluorophosphate, **HBTU-** N, N, N′, N′- Tetramethyl-O-(1H-benzotriazole-1-yl)uroniumhexafluorophosphate, **HCTU-** O-(1H-6-Chlorobenzotriazole-1-yl)-1,1,3,3-tetramethyluronium hexafluorophosphate, **HOBt-** 1-Hydroxybenzotriazolemonohydrate, **Hex**- Hexanes, **K_2CO_3** - Potassium carbonate, **MeOH**- Methanol, **NH_4Cl**- ammonium chloride, **$NaHCO_3$** - Sodium bicarbonate, **$Na_2S_2O_3$** - Sodium Thiosulfate, **Na_2SO_4** - Sodium Sulfate, **PABA-** p-aminobenzyl alcohol, **$Pd(PPh_3)_4$**-Tetrakis(triphenylphosphine)palladium(0), **TMS-Cl** - Trimethylsilyl chloride.

Protected amino acids used in peptides synthesis.

Fmoc-Gly-OH, Fmoc-Ala-OH, Fmoc-Val-OH, Fmoc-Leu-OH, Fmoc-Ile-OH, Fmoc-Phe-OH, Fmoc-His(Trt)-OH, Fmoc-Asn(Trt)-OH, Fmoc-Gln(Trt)-OH, Fmoc-Arg(Pbf)-OH, FmocLys(Boc)-OH, Fmoc-Tyr(tBu)-OH, Fmoc-Ser(tBu)-OH, Fmoc-Thr(tBu)-OH, Fmoc-Asp(OtBu)- OH, Fmoc-Glu(OtBu)-OH, Fmoc-Lys(Alloc)-OH, Boc-Cys(Trt)-OH, Fmoc-Nle-OH, Fmoc-ValThr(ψMe,MePro)-OH, Fmoc-Ile-Thr(ψMe,MePro)-OH, Fmoc-Glu(tBu)-Thr(ψMe,MePro)-OH, FmocIle-Ser(ψMe,MePro)-OH, Fmoc-Asp(OtBu)-(DMB)Gly-OH. N-methyl-3,4-diaminobenzoic acid (Fmoc-NMeDbz),[**1**] was prepared using the reported procedure.

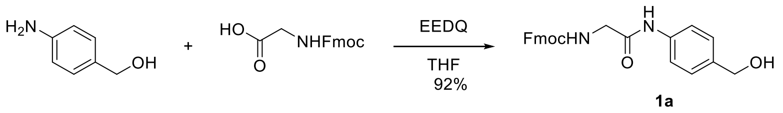

Compound 1a

PABA (200 mg, 1.6 mmol, 1 eq) was dissolved in 4 ml of THF. Fmoc-Gly-OH (480 mg, 1.6 mmol, 1 eq) was added followed by the addition of EEDQ (610 mg, 2.4 mmol, 1.5 eq). The reaction was monitored by TLC (50:50 EtOAc:Hex). Upon completion, the solvent was removed by reduced pressure. The resulting mixture was precipitated and washed with cold diethyl ether solution affording Compound 1a (600 mg, 92% yield) as a yellow solid. ^{1}H NMR (400 MHz, DMSO) δ 9.89 (s, 1H), 7.88 (d, J = 7.5 Hz, 2H), 7.71 (d, J = 7.4 Hz, 2H), 7.59 (t, J = 6.0 Hz, 1H), 7.51 (d, J = 8.4 Hz, 2H), 7.40 (t, J = 7.4 Hz, 2H), 7.32 (t, J = 7.4 Hz, 2H), 7.22 (d, J = 8.4 Hz, 2H), 5.06 (s, 1H), 4.41 (s, 2H), 4.26 (dd, J = 22.5, 6.7 Hz, 3H), 3.77 (d, J = 6.1 Hz, 2H).^{13}C NMR (100 MHz, DMSO) δ 168.33, 144.42, 141.30, 138.07, 128.19, 127.64, 127.52, 125.81, 120.67, 119.38, 66.30, 63.17, 47.21, 44.56.

MS (ES+): m/z calc. for C24H22N2O4: 402.1; found: 425.3 [M+Na]$^+$.

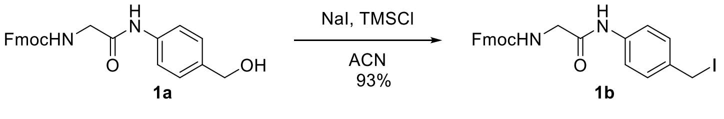

Compound 1b

Compound 1a (600 mg, 1.6 mmol, 1 eq) was dissolved in 10 ml of ACN. Sodium Iodide (640 mg, 4.8 mmol, 3 eq) was added followed by the rapid addition of TMS-Cl (540 µl, 4.8 mmol, 3eq). The reaction was monitored by TLC (50:50 EtOAc:Hex). Upon completion, the solvent was removed by reduced pressure. The resulting mixture was precipitated and washed with cold diethyl ether solution affording Compound 1b (740 mg, 93% yield) as a yellow solid. ^{1}H NMR (400 MHz, DMSO) δ 10.33 (s, 1H), 7.87 (d, J = 7.5 Hz, 2H), 7.71 (d, J = 7.4 Hz, 2H), 7.65 – 7.57 (m, 3H), 7.41 – 7.26 (m, 6H), 4.69 (s, 2H), 4.25 (dd, J = 19.2, 6.4 Hz, 3H), 3.81 (d, J = 5.7 Hz, 2H). ^{13}C NMR (100 MHz, DMSO)

δ 168.69, 157.16, 144.39, 141.28, 139.60, 132.77, 130.04, 128.20, 127.64, 125.82, 120.68, 119.63, 66.33, 65.46, 47.19, 46.78, 44.64.

MS (ES+): m/z calc. for C24H21IN2O3: 512.1 ; found: 535.2 $[M+Na]^+$.

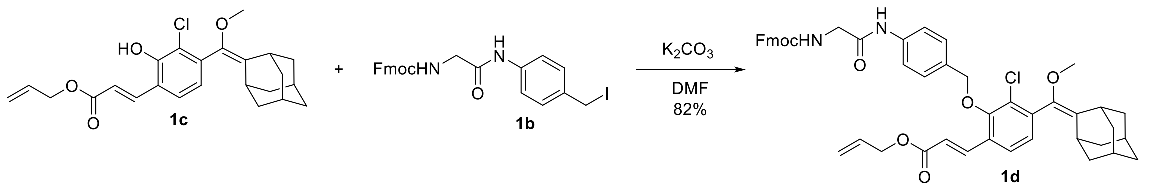

Compound 1d

Compound 1c[1] (60 mg, 0.1 mmol, 1 eq) and K_2CO_3 (30 mg, 0.15 mmol, 1.5 eq) were dissolved in dry DMF and stirred for 10 minutes. Then, compound 1b (74 mg, 0.1 mmol, 1 eq) was added and the reaction was stirred at 40°C and monitored by RP-HPLC (70-100% ACN in water, 20 min). Upon completion, the reaction mixture was diluted with EtOAc and washed with 1M HCl followed by brine. The organic layer was separated, dried over Na_2SO_4, filtered and the solvent was evaporated under reduced pressure. The product was purified by column chromatography on silica gel (50:50 EtOAc:Hex) to afford compound 1d (90 mg, 82% yield) as an off-white solid. ^{1}H NMR (400 MHz, $CDCl_3$) δ 8.33 (s, 1H), 7.94 (d, J = 16.2 Hz, 1H), 7.75 (d, J = 7.5 Hz, 2H), 7.58 (d, J = 7.3 Hz, 2H), 7.52 (d, J = 8.3 Hz, 2H), 7.45 – 7.34 (m, 6H), 7.33 – 7.26 (m, 2H), 7.07 (d, J = 8.0 Hz, 1H), 6.45 (d, J = 16.2 Hz, 1H), 6.03 – 5.91 (m, 1H), 5.78 (s, 1H), 5.40 – 5.23 (m, 2H), 4.96 (d, J = 3.4 Hz, 2H), 4.72 – 4.66 (m, 2H), 4.46 (d, J = 6.9 Hz, 2H), 4.22 (t, J = 6.9 Hz, 1H), 4.02 (s, 2H), 3.32 (s, 3H), 3.28 (s, 1H), 2.08 (s, 1H), 1.84 (ddd, J = 33.3, 27.7, 12.0 Hz, 12H).^{13}C NMR (101 MHz, CDCl3) δ 167.27, 166.41, 157.08, 153.77, 143.70, 141.42, 139.53, 139.13, 138.35, 137.82, 132.55, 132.30, 129.85, 129.77, 127.91, 127.23, 125.16, 125.07, 120.14, 119.99, 118.35, 75.79, 67.54, 65.38, 60.51, 57.34, 47.18, 45.63, 39.30, 39.15, 38.72, 37.16, 33.06, 32.02, 31.72, 29.82, 28.46, 28.33, 22.78, 21.13, 14.28.

MS (ES+): m/z calc. for C48H47ClN2O7: 798.9; found: 800.1 $[M+H]^+$.

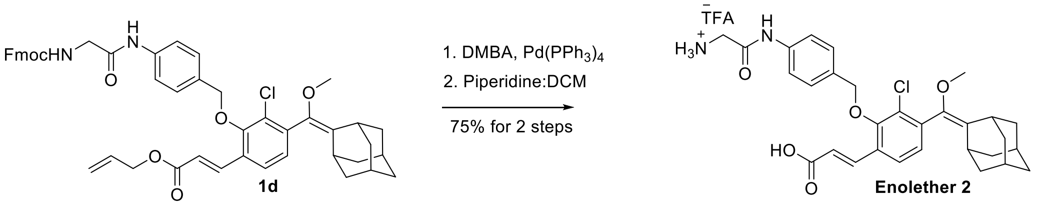

Enolether 2

A mixture of **compound 1d** (40 mg, 0.05 mmol, 1 eq), DMBA (23 mg, 0.15 mmol, 3 eq) and $Pd(PPh_3)_4$ (18 mg, 0.3 eq) in DCM (2 mL) was stirred at room temperature for 3 hours and monitored by RP-HPLC (70-100% ACN in water, 20 min). Upon full consumption of starting material, 0.5 ml of piperidine was added. The reaction was

monitored by RP-HPLC (70-100% ACN in water, 20 min). Upon completion, the reaction mixture was concentrated under reduced pressure and the crude product was purified by preparative RP-HPLC (30-100% ACN in water, 20 min) to afford Enolether 2 (20 mg, 75% yield) as a white solid. ^{1}H NMR (400 MHz, DMSO) δ 10.44 (s, 1H), 7.76 (dd, J = 12.1, 4.0 Hz, 2H), 7.57 (t, J = 8.0 Hz, 2H), 7.42 (d, J = 8.5 Hz, 2H), 7.08 (t, J = 13.9 Hz, 1H), 6.57 (d, J = 16.1 Hz, 1H), 5.01 – 4.84 (m, 2H), 3.75 (s, 2H), 3.21 (s, 3H), 3.18 (d, J = 5.6 Hz, 1H), 1.80 (ddd, J = 47.1, 35.1, 20.9 Hz, 13H). ^{13}C NMR (101 MHz, DMSO) δ 167.93, 165.52, 153.41, 139.98, 138.95, 137.76, 132.06, 131.85, 130.80, 130.13, 129.25, 128.32, 126.46, 122.32, 120.54, 119.52, 75.73, 57.12, 42.70, 41.71, 40.75, 40.54, 40.33, 40.12, 39.91, 39.71, 39.06, 38.75, 37.06, 33.01, 29.63, 28.72, 28.29.

MS (ES+): m/z calc. for C30H33ClN2O5: 536.2; found: 537.4 $[M+H]^+$.

Compound 1 (Ub(1-75)-MeNbz)

Compound 1 was synthesized on Rink amide resin (0.26 mmol/g, 0.1 mmol scale). Fmoc-MeDbz (4 equiv.) was coupled to the pre-swollen resin using HATU (4 equiv.), DIEA (8 equiv.) for 90 min. The Fmoc was then deprotected by treatment of the resin with 20% piperidine for three cycles of 3, 5, and 3 min each. The C-terminal residue of Gly75 was coupled using HCTU (4 equiv.) and DIEA (8 equiv.) for 45 min (2 cycles). The remaining amino acids were coupled by standard Fmoc-SPPS on the synthesizer. After completion of SPPS, the peptide on the resin (0.1 mmol) was treated with p-nitrophenyl chloroformate (100 mg, 5 equiv.) in 4 ml dry DCM for 30 min (3 cycles), and was washed with DCM and DMF, followed by treatment in 4 ml of 0.5 M DIEA in DMF for 10 min (3 cycles). After this, the peptide was cleaved using TFA:TIS:water (95:2.5:2.5) cocktail (9 mL for 0.025 mmol peptide resin) for 2 h to give **Compound 1 (Ub(1-75)-MeNbz)**. The cleavage mixture was filtered dropwise to a 10-fold volume of cold ether and centrifuged. The precipitated crude peptide was dissolved in acetonitrile-water (1:1) and lyophilized. The crude peptide 3 was purified by preparative HPLC using a C4 column with a gradient of 0-60% buffer B over 30 min to afford the corresponding peptide **Compound 1** in ~22 % isolated yield.

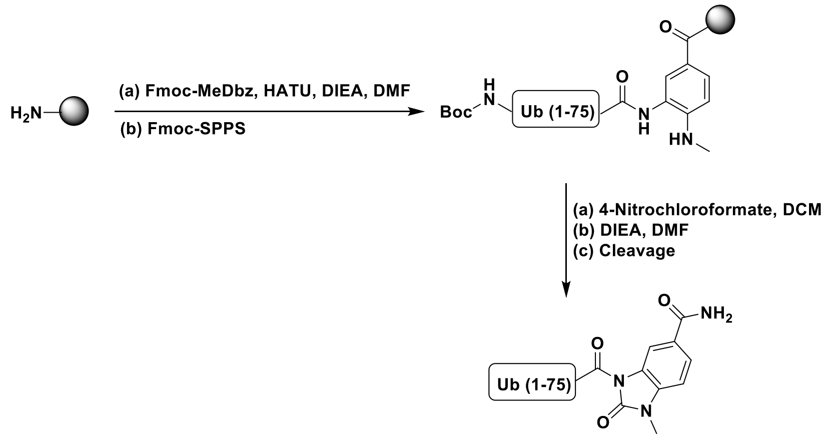

(1)

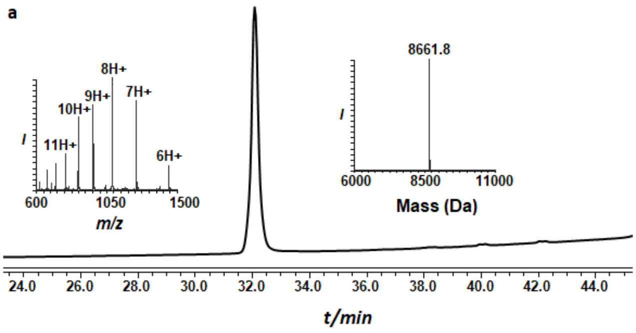

Figure S1. Synthesis of **Compound 1** (Ub(1-75)-MeNbz): (a) Analytical HPLC and mass analysis of purified **1** with the observed mass 8661.8 ± 0.3 Da, calcd 8662.7 Da (average isotopes). Detection of HPLC chromatogram at 214nm.

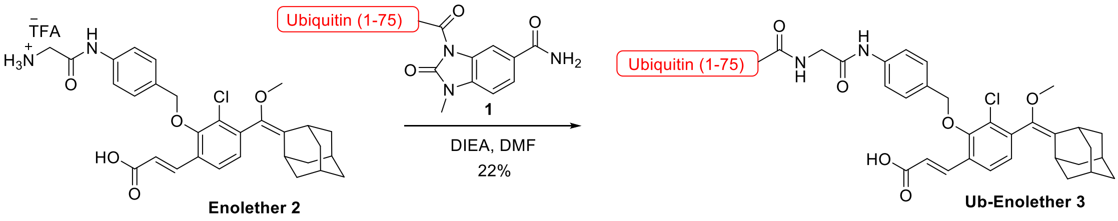

Ub-Enolether 3

To a solution of **Enolether 2** (25 eq) in DMF was added DIEA (10 eq) and incubated at room temperature for 30 min. After 30 min, the solution was added to ubiquitin-Nbz (**1**, 1 eq) and incubated for another 24 h. The reaction mixture was diluted with acetonitrile:water and lyophilized. The lyophilized crude product was purified using a C18 semi-preparative column with the gradient flow of 0-60% ACN over 41 min and purified ubiquitin-enol ether (**Ub-Enolether 3**) was obtained in 22% isolated yield.

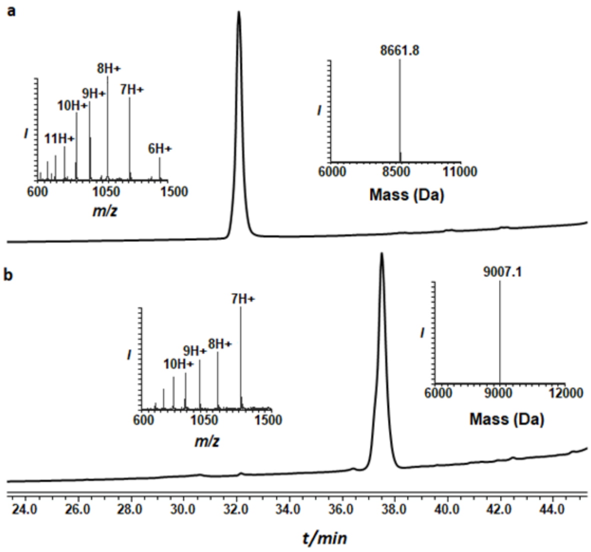

Figure S2. Synthesis of **Ub-Enolether 3**: (a) Analytical HPLC and mass analysis of purified **1** with the observed mass 8661.8 ± 0.3 Da, calcd 8662.7 Da (average isotopes). (b) Analytical HPLC and mass analysis of purified **Ub-Enolether 3** with the observed mass 9007.1 ± 0.2 Da, calcd 9008.7 Da (average isotopes). Detection of HPLC chromatogram at 214nm.

Ub-Enolether 3 Oxidation to Ub-CL– Procedure

Method 1: To a 20 ml vial containing 0.5 mg/ml of **Ub-Enolether 3** in TRIS, pH 7.5, were added 34 mg of Polystyrene-bound Rose Bengal (0.1 mmol/g). The vial was cooled in ice water bath and oxygen was vigorously bubbled through while irradiating with white light for 20 minutes. The solution of probe **Ub-CL** was filtered and further used as a stock solution for measurements.

Method 2: To a 20 ml vial containing 0.5 mg/ml of **Ub-Enolether 3** in DMF, was added catalytic amount of Methylene Blue (~ 0.1 mg). The vial was cooled in ice water bath and oxygen was vigorously bubbled through while irradiating with white light for 3 minutes. The probe **Ub-CL** was purified by RP-HPLC (10-90% ACN).

A

B

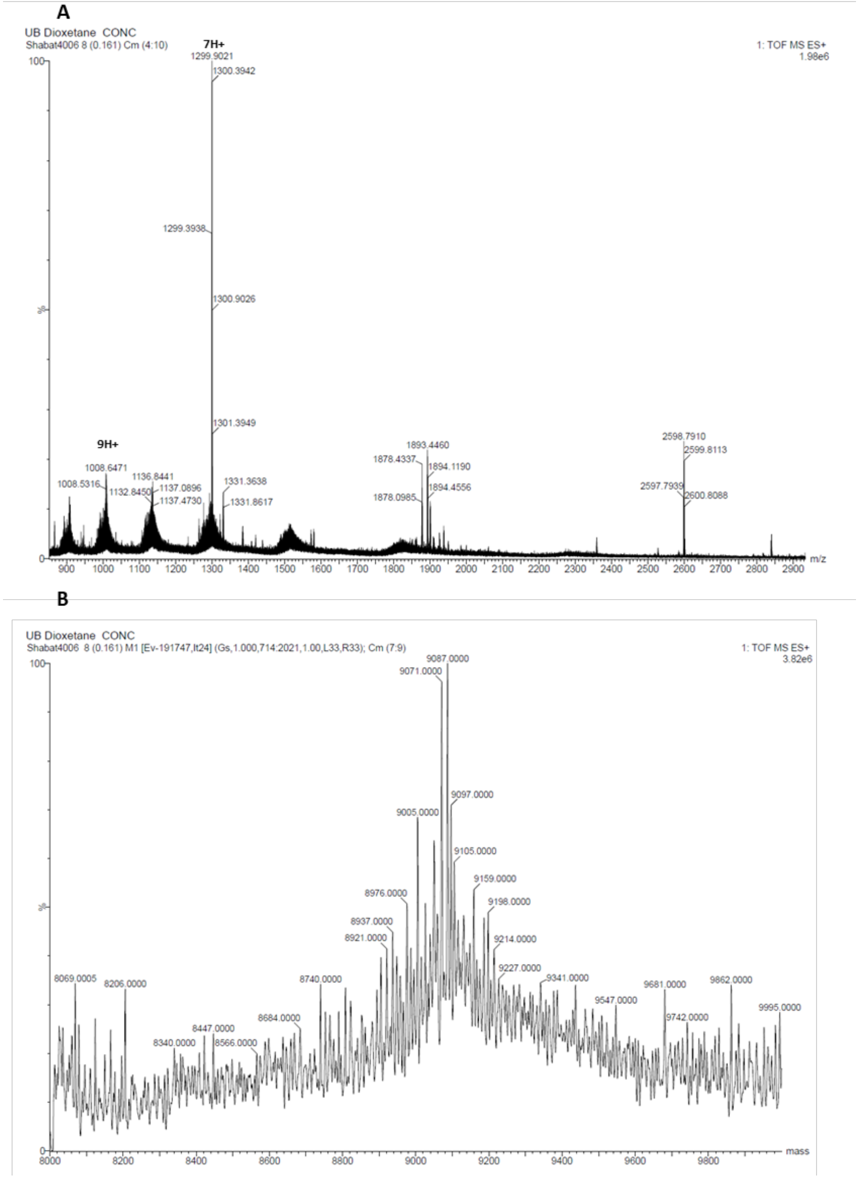

C.

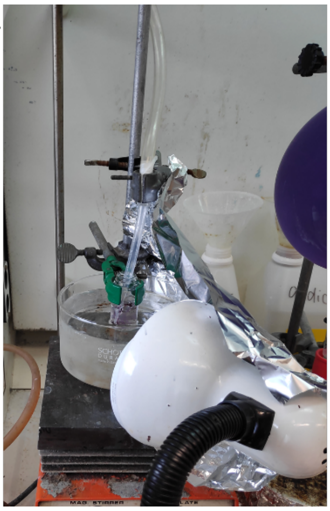

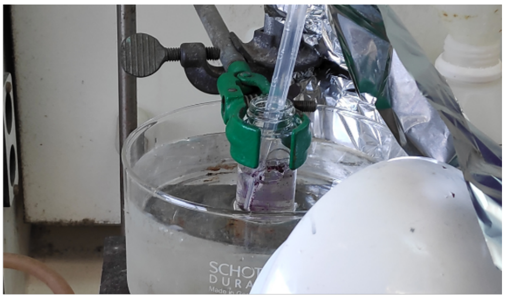

Oxidation setup:

Cooling bath
Lamp
Oxygen (cylinder)

Figure S3. A. Mass analysis of purified **Ub-CL** with the observed mass (1299.9021 [M+7H]/7 and 1008.6471 [M+9H]/9) **B.** Mass analysis of purified **Ub-CL** with the calculated mass by average isotopes (9071.00). **C.** Pictures illustrating the oxidation reaction setup.

Supplementary Figures

Figure S4. Oxidation in TRIS, 7.5 pH: Kinetic profile of Ub-CL upon reaction with UCH-L3 [80 nM] after oxidation duration of 8 minutes.

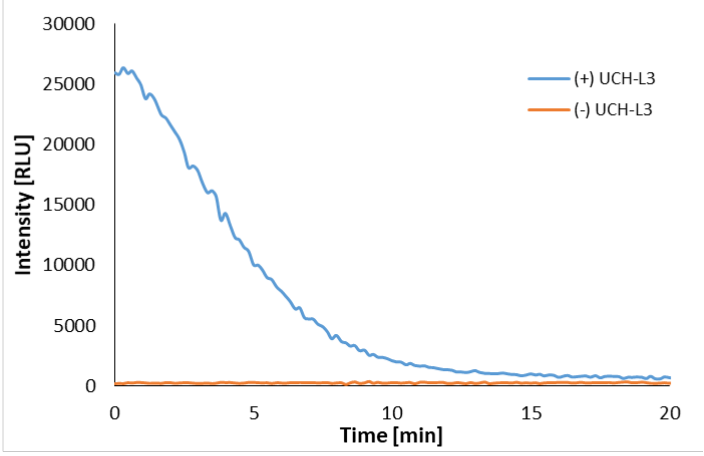

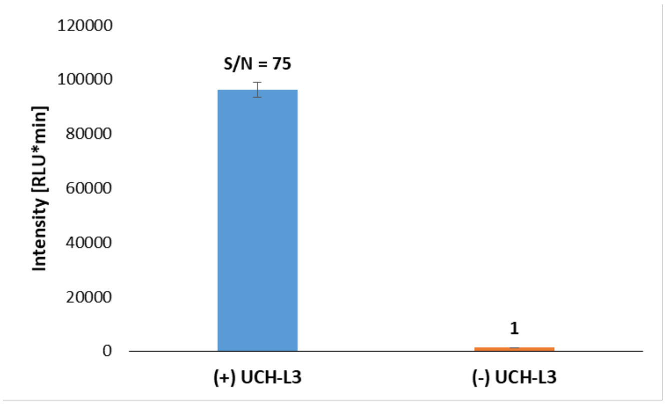

Chemiluminescence kinetic profile and Total light emission of Ub-CL [50 nM] in TRIS PH 7.5, DTT [0.2 mM] with and without UCH-L3 [80 nM]. The total light emission was measured after 5 minutes at 37° C. The signal to noise ratio measured at T_{max} is 128.

Figure S5. Oxidation in TRIS, 7.5 pH: Kinetic profile of Ub-CL upon reaction with UCH-L3 [8 nM] after oxidation duration of 8 minutes.

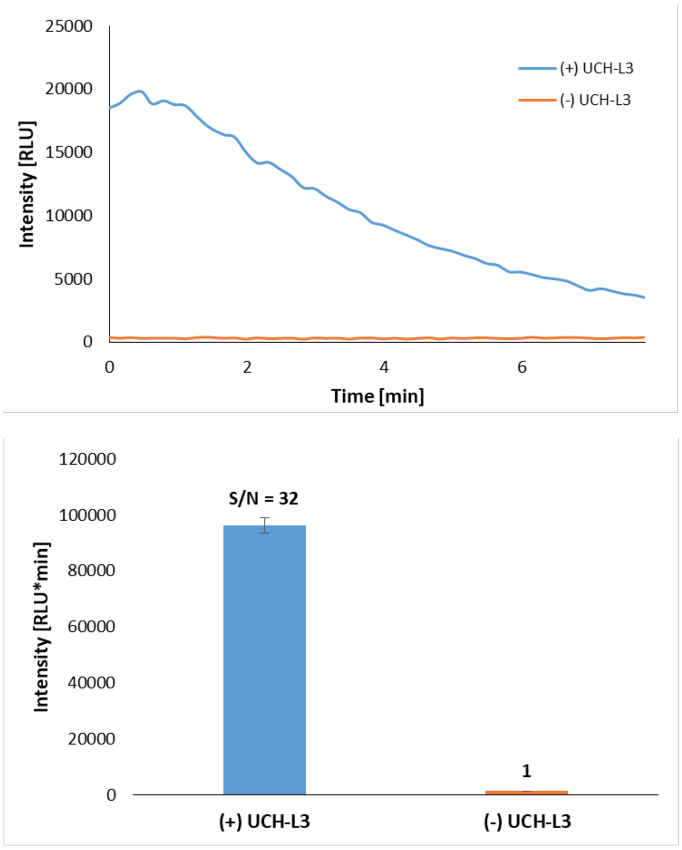

Chemiluminescence kinetic profile and Total light emission of Ub-CL [50 nM] in TRIS PH 7.5, DTT [0.2 mM] with and without UCH-L3 [8 nM]. The total light emission was measured after 5 minutes at 37° C. The signal to noise ratio measured at T_{max} is 67.

Figure S6. Full kinetic profile of Ub-CL upon reaction with USP-2 [12 nM].

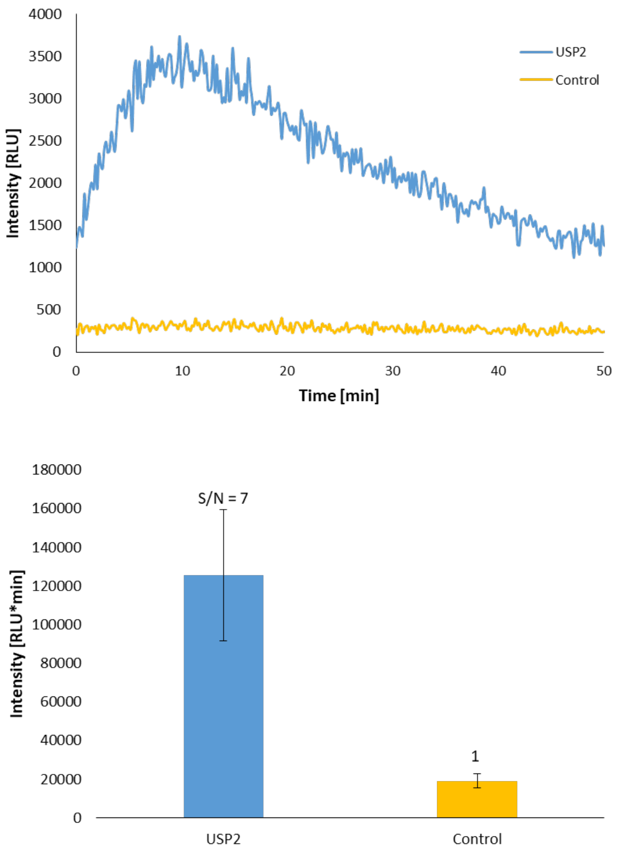

Chemiluminescence kinetic profile and Total light emission of Ub-CL [50 nM] in TRIS PH 7.5, DTT [0.2 mM] with and without USP-2 [12nM]. The total light emission was measured after 50 minutes at 37° C.

Figure S7. Stability measurements of Ub-CL in TRIS, 7.5 pH, upon reaction with UCH-L3 [0.8 nM] after one, two, three days, and one month of storage.

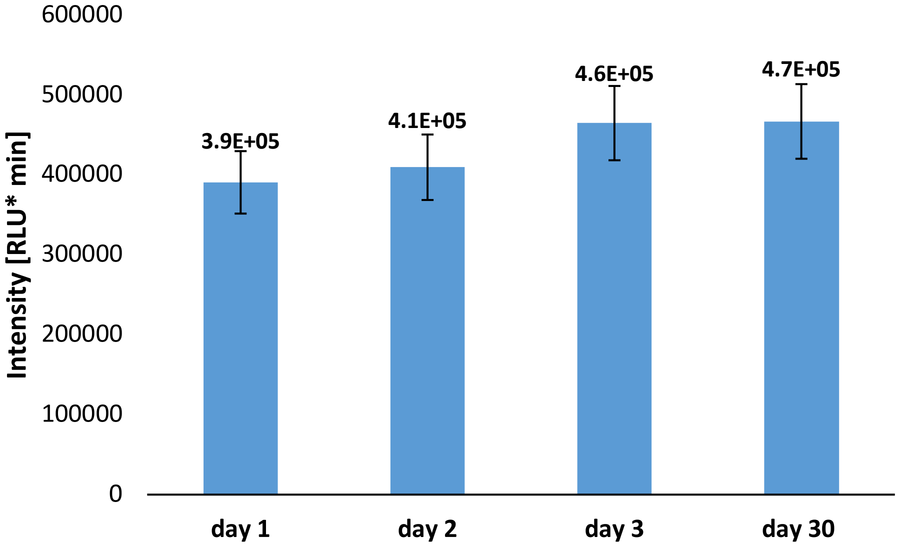

Chemiluminescence total light emission of Ub-CL [50 nM] in TRIS PH 7.5, DTT [0.2 mM] with and without UCH-L3 [0.8 nM]. The total light emission was measured after 15 minutes at 37° C.

Figure S8. Concentration calibration curve of a known chemiluminescent probe (TPCL).[2,3]

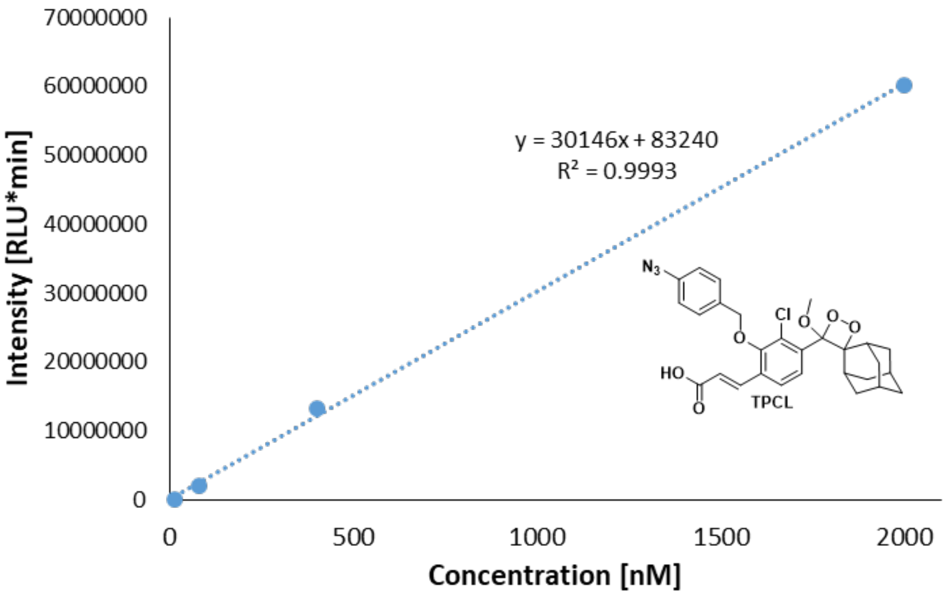

Concentration [nM]	Total Light Emission	STDEV
2000	60174632.6	1660047.2
400	13253397.4	504253.8
80	2064076.6	94907.6
16	86078.2	26192.1

Chemiluminescence total light emission as function of various concentrations of TPCL with and without Triphenyl Phosphine [10 eq] allows extrapolation of Ub-CL, as measured in TRIS PH 7.5, DTT[0.2 mM] with and without UCH-L3 [0.8 nM].

Figure S9. Total light emission as a function of time of Ub-CL (left) and Ub-AMC (right) in TRIS, 7.5 pH, upon reaction with or without of UCH-L3 [0.8 nM].

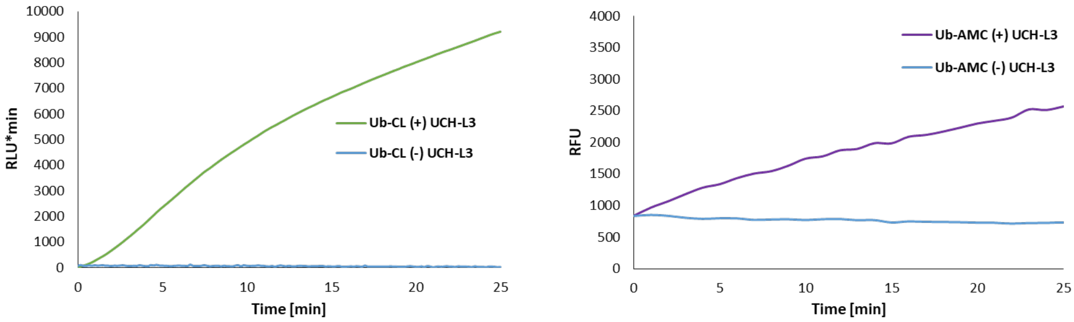

Figure S9. Chemiluminescence total light emission (left) and fluorescence (right) kinetic profiles of Ub-CL [50 nM] and Ub-AMC [1 uM] in TRIS PH 7.5, DTT [0.2 mM] at 37°C with and without UCH-L3 [0.8 nM]

NMR spectra

^{1}H-NMR (Compound 1a)

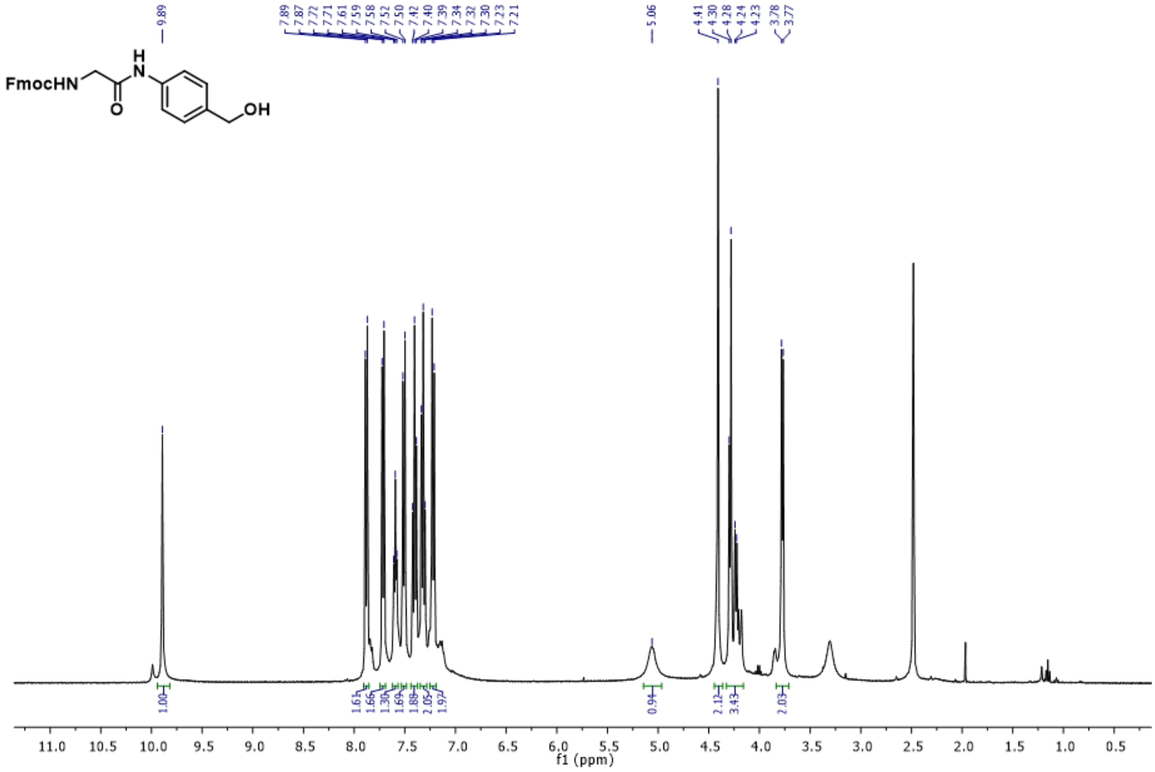

^{13}C-NMR (Compound 1a)

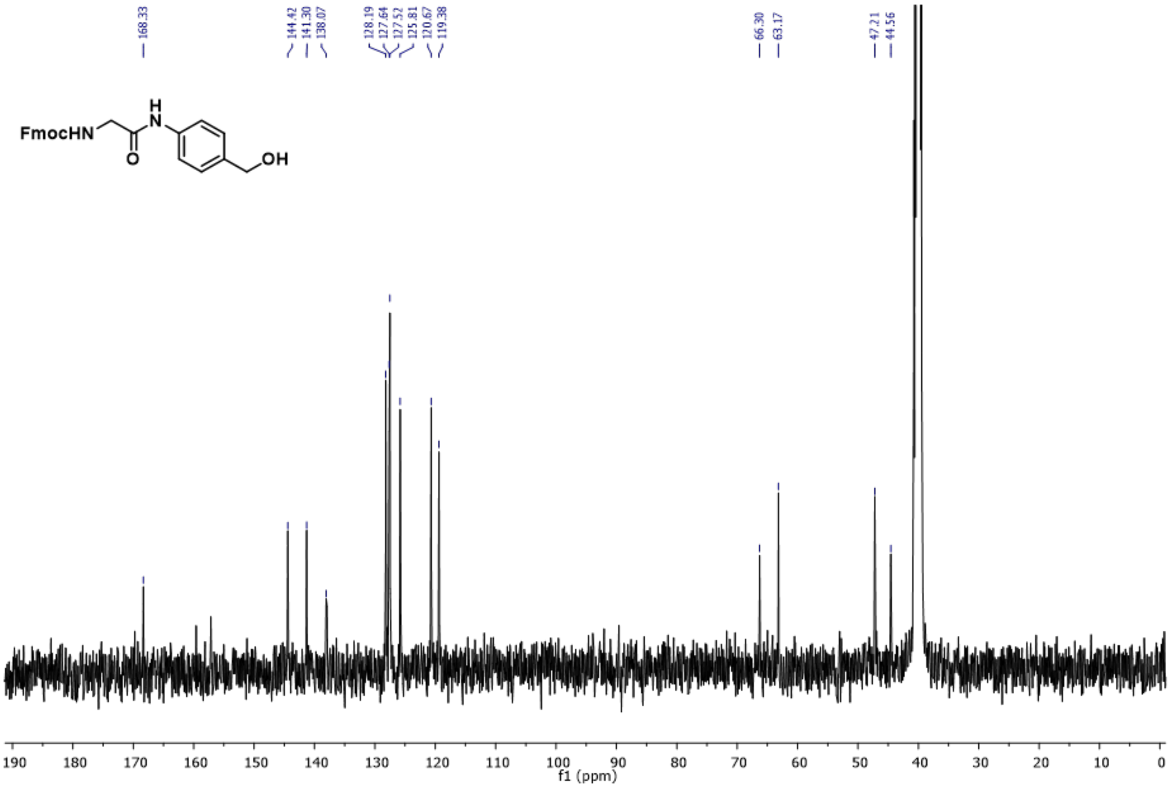

^{1}H-NMR (Compound 1b)

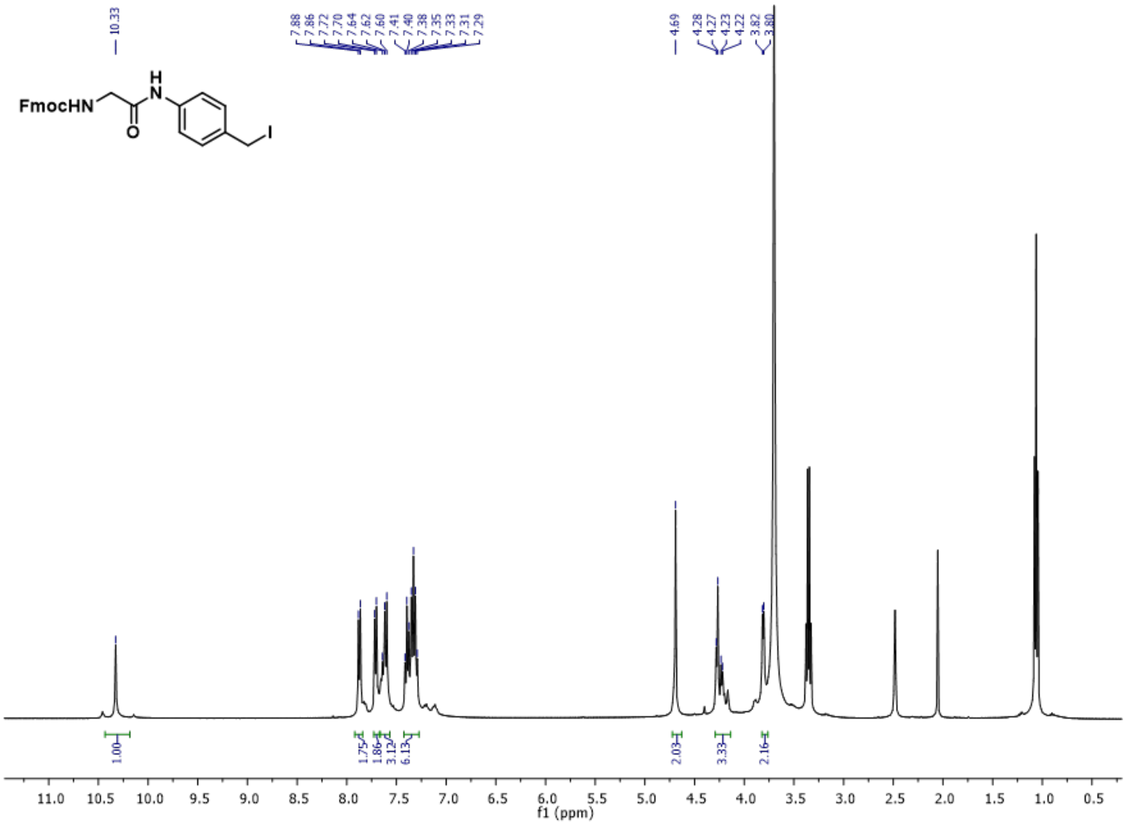

^{13}C-NMR (Compound 1b)

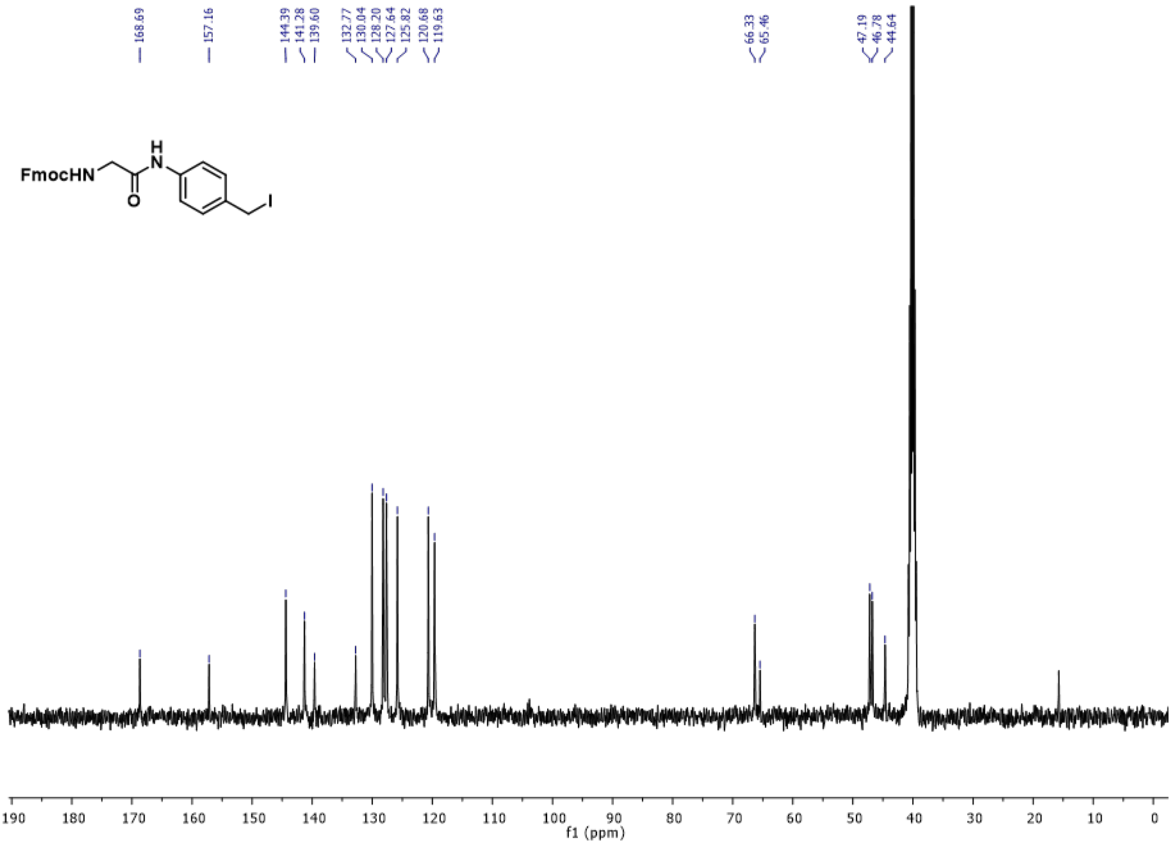

^{1}H-NMR (Compound 1d)

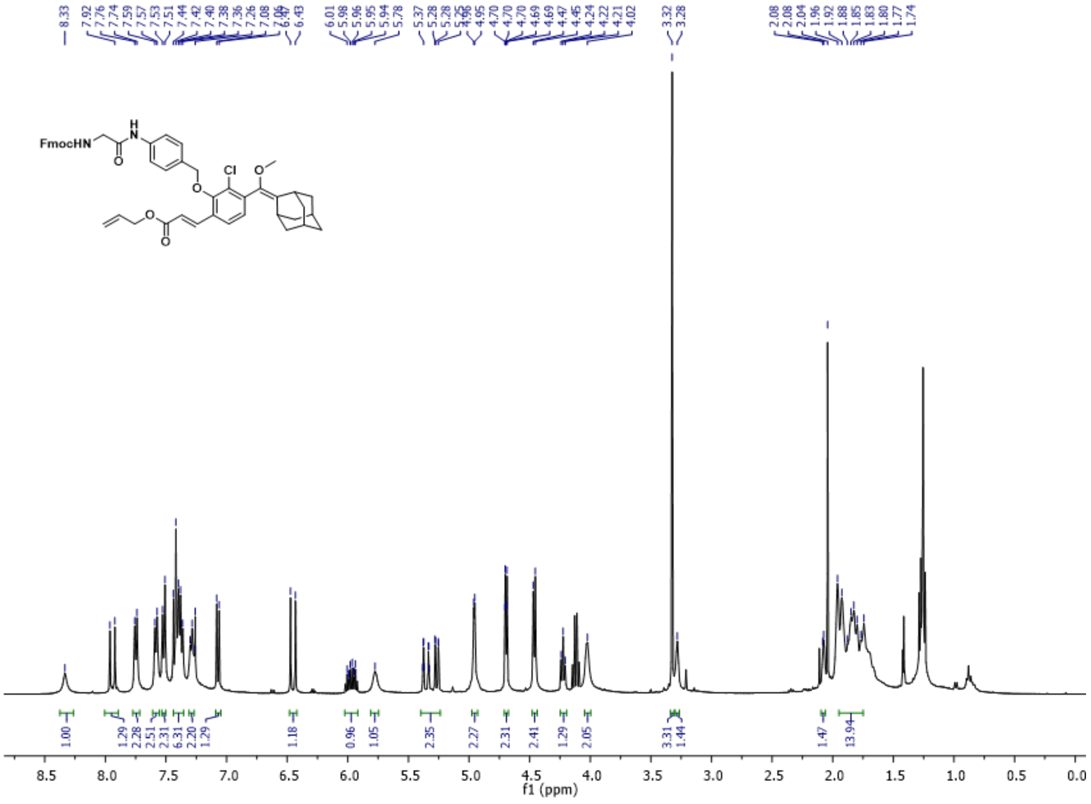

^{13}C-NMR (Compound 1d)

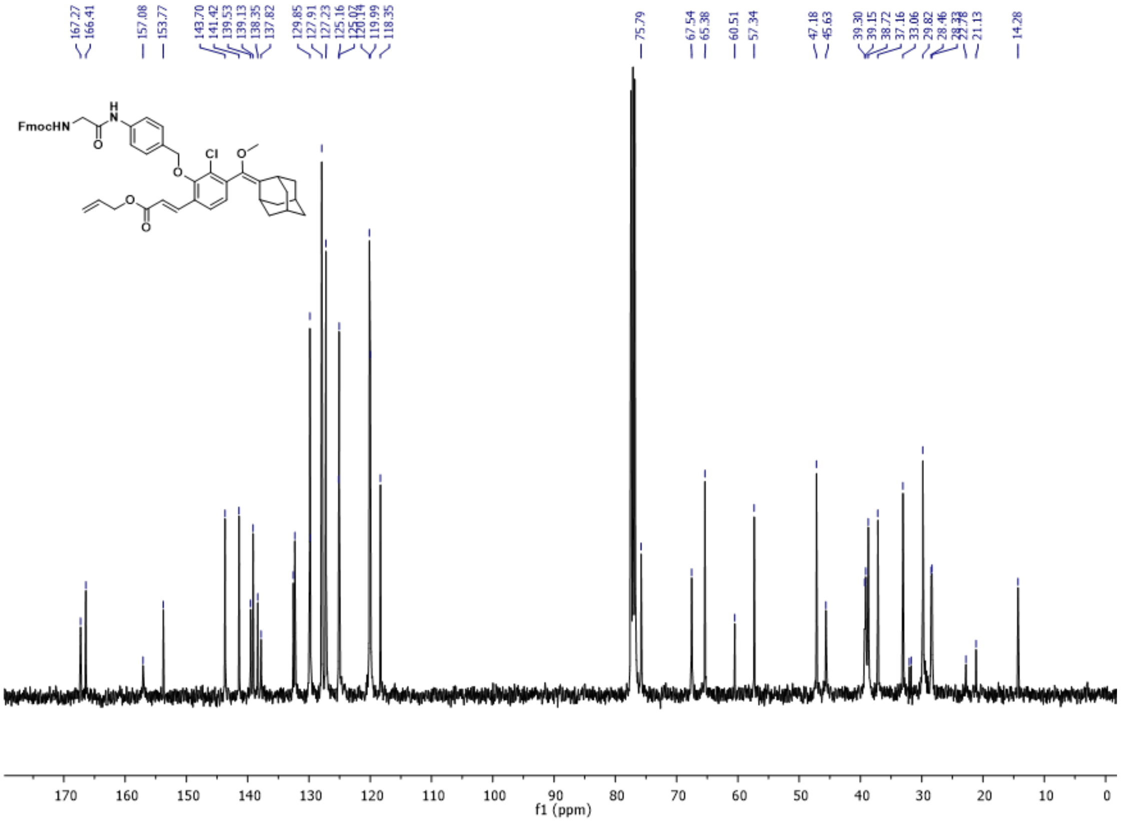

^{1}H-NMR (Enolether 2)

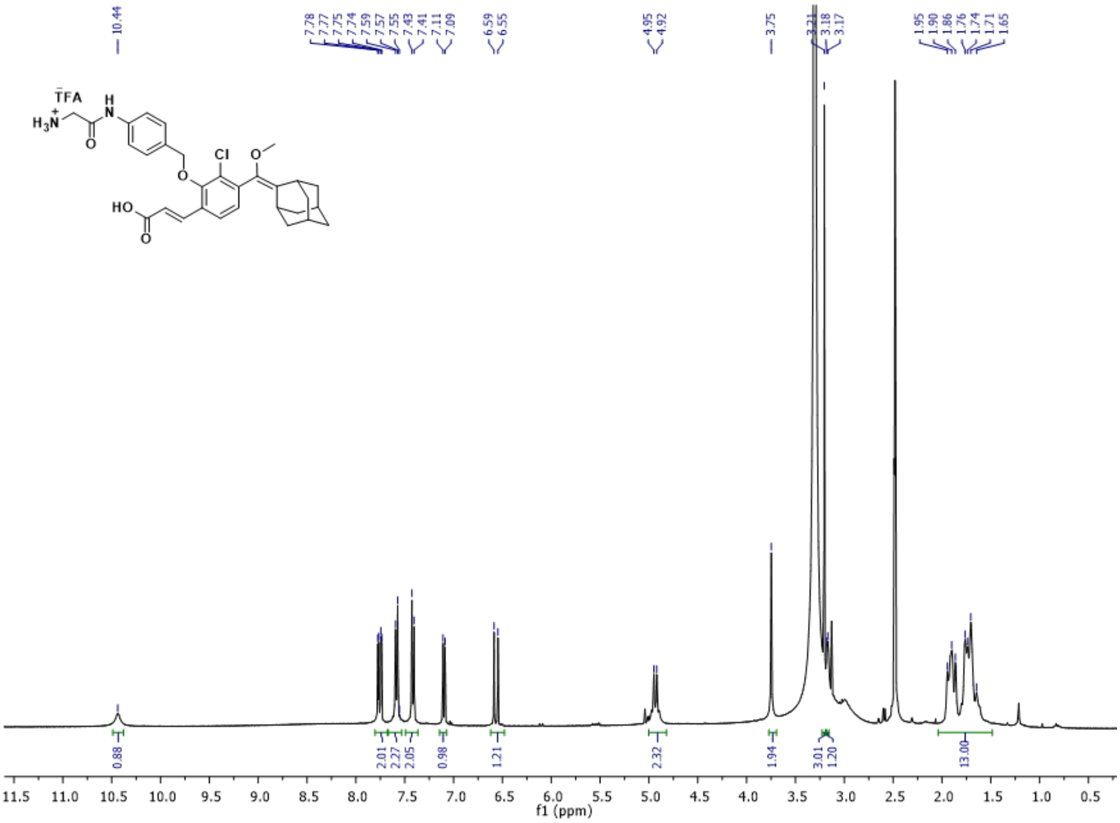

^{13}C-NMR (Enolether 2)

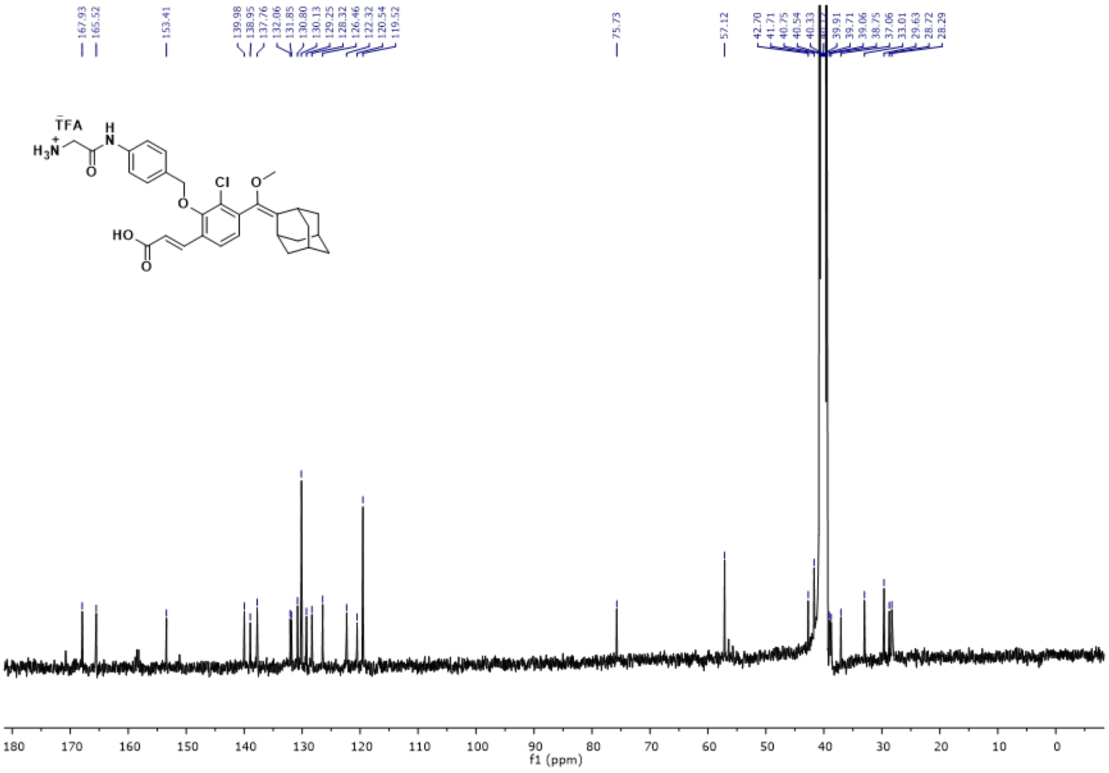

References

(1) Hananya, N.; Reid, J. P.; Green, O.; Sigman, M. S.; Shabat, D. (2019) Rapid Chemiexcitation of Phenoxy-Dioxetane Luminophores Yields Ultrasensitive Chemiluminescence Assays. *Chem. Sci. 10* (5), 1380–1385.

(2) J. Cao; R. Lopez; M. Thacker, J.; Y. Moon, J.; C. Jiang; S. Morris, S. N.; H. Bauer, J.; P. Tao; P. Mason, R.; R. Lippert, A. (2015) Chemiluminescent Probes for Imaging H 2 S in Living Animals. *Chem. Sci. 6* (3), 1979–1985.

(3) Luo, J.; Liu, Q.; Morihiro, K.; Deiters, A. (2016) Small-Molecule Control of Protein Function through Staudinger Reduction. *Nat. Chem. 8* (11), 1027–1034.
